# Comparison of bacterial diversity in *Bactrocera cucurbitae* (Coquillett) ovaries and eggs based on 16S rRNA sequencing

**DOI:** 10.1038/s41598-023-38992-z

**Published:** 2023-07-21

**Authors:** Chen Lixiang, Tian Zhenya, Ma Weihua, Wang Jingjing, Huang Qiaofen, Zhou Yongping, Gao Xuyuan, Chen Hongsong, Zhou Zhongshi

**Affiliations:** 1 State Key Laboratory for Biology of Plant Diseases and Insect Pests, Institute of Plant Protection, Chinese Academy of Agricultural Sciences, Beijing, 100193 China; 2 National Nanfan Research Institute, Chinese Academy of Agricultural Sciences, Sanya, 572019 China; 3 College of Plant Science and Technology, Huazhong Agricultural University, Wuhan, 430070 China; 4 Guangxi Key Laboratory for Biology of Crop Diseases and Insect Pests, Institute of Plant Protection, Guangxi Academy of Agricultural Sciences, Nanning, 530007 China

**Keywords:** Microbiology, Bacteria, Metagenomics

## Abstract

Next-generation sequencing allows for fine-scale studies of microbial communities. Herein, 16S ribosomal RNA high-throughput sequencing was used to identify, classify, and predict the functions of the bacterial communities in the eggs and ovaries of *Bactrocera cucurbitae* (Coquillett) (Diptera: Tephritidae), which is a pest that infests a variety of cucurbit fruits at different developmental stages. Taxonomic analyses indicate that bacteria associated with *B. cucurbitae* represent 19 phyla, which were spread across different developmental stages. Specifically, the egg microbiota had a higher alpha diversity than those of microbiota in the primary and mature ovaries. Significant differences were not observed between the primary and mature ovaries in terms of their microbiota’s alpha diversities. Pseudomonadota, Deinococcota, Bacteroidota, Bacillota, and Actinomycetota were the dominant phyla in all three developmental stages of *B. cucurbitae*, and *Pseudomonadaceae* and *Enterobacteriaceae* were the most abundant families. Owing to the unique physiological environment of the ovaries, the diversity of their bacterial community was significantly lower than that in the eggs. This study provides new insights into the structure and abundance of the microbiota in *B. cucurbitae* at different developmental stages and contributes to forming management strategies for this pest.

## Introduction

Insects and microbes have established a variety of symbiotic relationships that have played important roles in their diversity and evolution^1–3^. Microorganisms participate in detoxification^4,5^, development^6,7^, physiology^8,9^, pathogen resistance^10^, immune response^11^, and essential vitamin and amino acid production^12,13^ in insects. Therefore, the study of microorganisms is important for investigating insect biology at a fine scale.

Insects harbor a range of microorganisms in their guts, ovaries, haemocoels, exoskeletons, and cells, among others. Throughout evolutionary history, these interactions have yielded interdependence between insects and various microbiota^14^. Petri^13^ provided the first report of bacterial symbionts in olive flies (*Bactrocera oleae*). Further studies of the symbiotic relationships between bacteria and flies have been conducted by investigating different fly organs, including the esophageal bulb and reproductive organs^15–19^. To date, many bacterial families have been observed in association with live flies, including Enterobacteriaceae, Bacillaceae, Pseudomonadaceae, Phyllobacteriaceae, Micrococcaceae, Erwiniaceae, Xanthomonadaceae, Acetobacteraceae, Lactobacillaceae, Pectobacteriaceae, Flavobacteriaceae, Streptococcaceae, Halomonadaceae, Brucellaceae, Yersiniaceae, Staphylococcaceae, Xanthomonadaceae, and Comamonadaceae^20–27^. These microorganisms can synthesize vitamins, nitrogen, and amino acids for their hosts and can be transmitted vertically from parents to the next generation^28^. For example, Burkholderiales and Opitutales microorganisms of genus *Cephalotes* can degrade nitrogenous waste (e.g., urea and uric acid) and use the products to synthesize large amounts of amino acids^29^. *Enterococcus* and *Pseudomonas* in *Spodoptera litura* contain enzyme-coding genes involved in cellulose, xylan, and pectin degradation that are important for facilitating polymer degradation by their hosts^30^. *Buchnera*, the endosymbiotic microorganism of *Acyrthosiphon pisum*, possesses most of the genes required for essential amino acid synthesis pathways, which its hosts lack^31^. In addition, bacteria have multiple applications in managing economically important fruit flies and other insect pests. For example, bacteria are associated with the degradation of toxic substances ingested by host insects and may contribute to pesticide resistance in host fruit flies^32^. Further, bacterium-linked odors have been reported to stimulate feeding or spawning in a variety of insects^20^, and bacteria are also used as bait or attractants in insect traps^33^. In addition, commensal bacteria are involved in the sterile insect technology project^34^. Bacteria can also impact the reproductive success of insects. For example, *Rickettsia* can increase the egg production and offspring survival rate of the whitefly and *Arsenophonus* may regulate the mating behavior of *Nasonia vitripennis*, thereby affecting the breeding dynamics of the host insect^35,36^.

The melon fruit fly *Bactrocera cucurbitae* is one of the most severe pests for a variety of horticultural crops. Specifically, it infests 81 different plant species and is a major pest of cucurbits, including bitter gourd (*Momordica charantia*), muskmelon (*Cucumis melo*), snap melon (*C. melo* var. *momordica*), and snake gourd (*Trichosanthes anguina*)^37^. *B. cucurbitae* can cause 30–100% crop losses depending on the cucurbit species and season^38,39^. Temperatures less than 32 °C and a relative humidity (RH) of 60–70% are ideal conditions for *B. cucurbitae* proliferation^39^. This insect oviposits in the tissue of the fruit at a depth of 2–4 mm. The hatched larvae feed on the fruit pulp and pupate 0.5–15 cm below the soil surface. The melon fruit fly is widely distributed throughout more than 30 temperate, subtropical, and tropical countries and regions and is believed to have originated in India^39^. *B. cucurbitae* is classified as one of the most important quarantine objects in numerous countries and regions^40^, and is an agriculturally significant insect species. Owing to its high adaptability and fecundity, *B. cucurbitae* requires novel approaches for pest management strategies.

Although *B. cucurbitae* is an important agricultural pest, few studies have focused on its microbiota. Previous studies have also largely utilized culture-dependent or low-resolution molecular techniques^25,41^. However, most research on *Zeugodacus tau* has focused primarily on its genetics or molecular phylogeny^42^, biology^43^, and behavior^44^. To date, the microbial association in *Z. tau* has only been investigated in adult flies^45–48^. To the best of our knowledge, previous studies have not reported on the bacteria associated with the ovaries and eggs (throughout different developmental stages) of *B. cucurbitae* using next-generation sequencing. Information regarding microbiota throughout a variety of developmental stages is also limited for other *Zeugodacus* fruit fly species, except for *Z. tau*^49^. Thus, in the present study, we used 16S ribosomal RNA (rRNA) gene sequencing with the Illumina HiSeq platform to determine the associations among various bacterial taxa in the eggs, primary ovaries, and mature ovaries of *B. cucurbitae*. We also predicted the functions of the bacterial communities in each of the sampled materials. The results of this study can be used to clarify the relative abundances and diversities of the microbiota associated with the eggs, primary ovaries, and mature ovaries of *B. cucurbitae*. In addition, the findings provide a basis for bacterium-linked *B. cucurbitae* management strategies.

## Results

### Analysis of taxa

**Figure 1:**
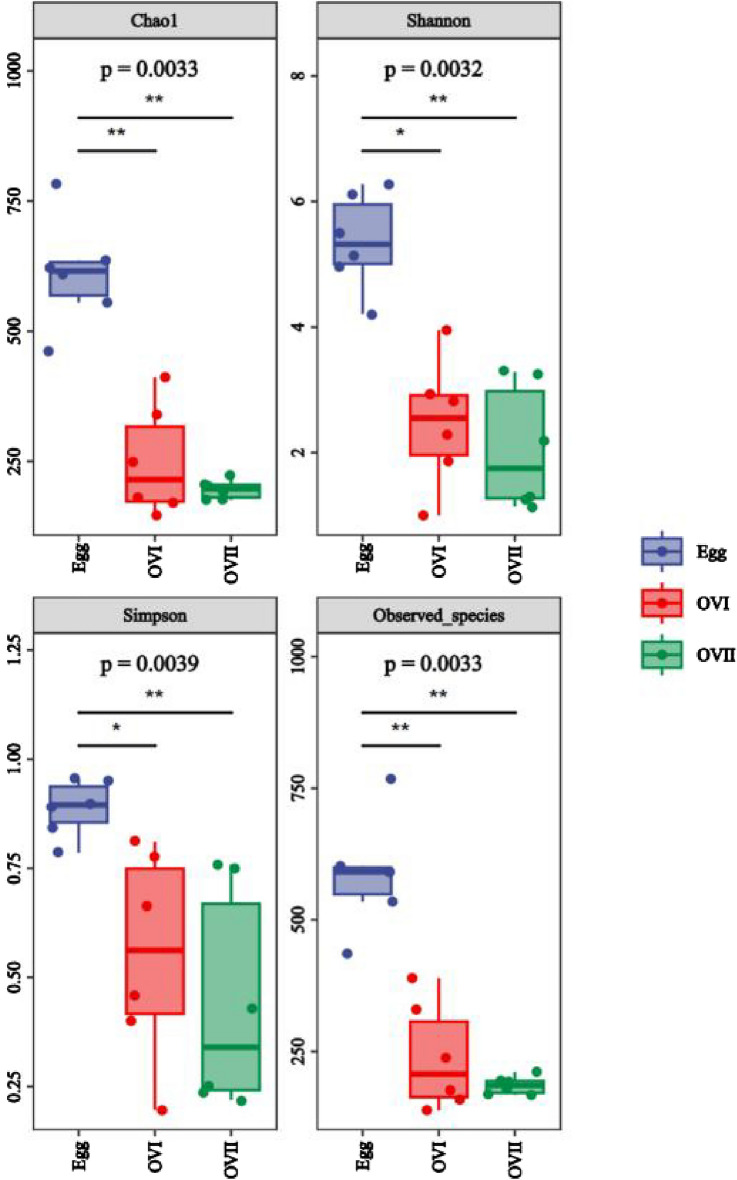
Boxplot of the Alpha diversity index. Coordinates are the grouping labels, the ordinate is the value of the corresponding alpha diversity index, and the numbers under the diversity index labels are the *P*-values of the Kruskal–Wallis test. Egg: Eggs of *Bactrocera cucurbitae*. OVI: Primary ovaries of *B. cucurbitae*. OVII: Mature ovaries of *B. cucurbitae*.

**Figure 2:**
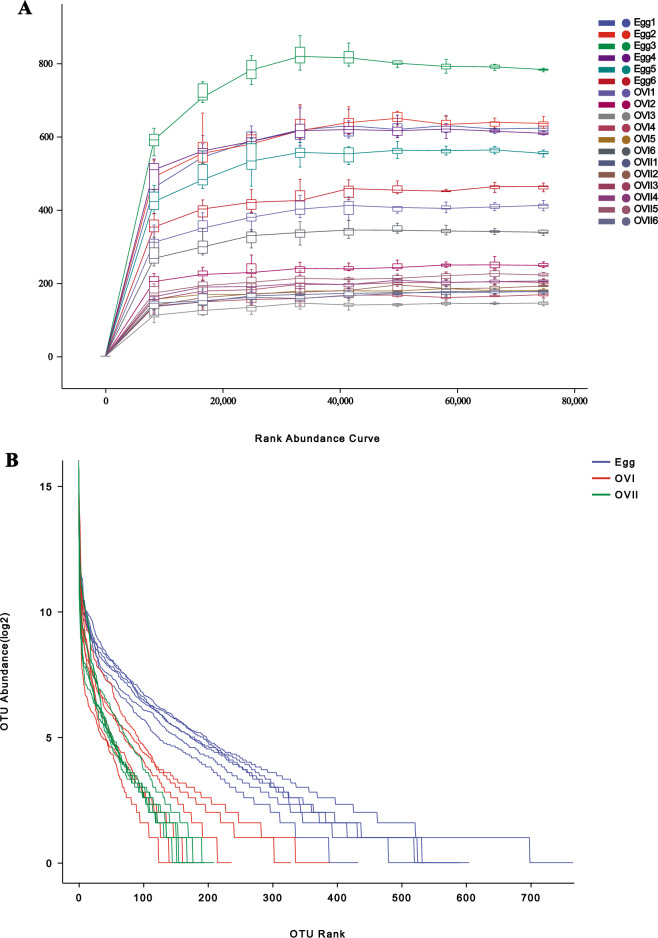
(**A**) Sample sparsity curves. The abscissa is the drawn leveling depth and the ordinate is the median value of the alpha diversity index calculated for 10 times and the boxplot. (**B**) Sample abundance rank curve. The abscissa is the ASV serial numbers arranged by abundance; the ordinate is the Log2 log-transformed abundance values of the ASVs in the sample/group. Egg1–egg6: Groups 1–6 samples were *B. cucurbitae* eggs. OVI1–OVI2: Groups 1–6 samples were *B. cucurbitae* primary ovaries. OVII1–OVII6: Groups 1–6 samples were *B. cucurbitae* mature ovaries.

We sequenced bacteria from the eggs, primary ovaries, and mature ovaries of *B. cucurbitae* and obtained 2,093,839 trimmed paired reads (Supplementary Table S1). The primer fragment of the QIIME cutadapt trim-paired excision sequence was called first, and the unmatched primers were discarded. DADA2 was called for quality control, denoising, splicing, and removal by QIIME DADA2 denoise pairing. The ranges of the Chao1, Simpson, and Shannon indices and the observed species were 175.912–782.66, 0.21922–0.955111, 1.13841–6.27746, and 0.21922–0.955111, respectively (Supplementary Table S2). Figure 1 shows a boxplot of the data. One-way analysis of variance (ANOVA) indicates that the alpha diversity indices differed significantly (*P* < 0.001) among the eggs, primary ovaries, and mature ovaries. However, the alpha diversity index did not differ markedly (*P* > 0.05) between the primary and mature ovaries (Supplementary Table S3). A total of 19 bacterial phyla, 39 classes, 73 orders, 151 families, 287 genera, and 376 species were identified in the eggs, primary ovaries, and mature ovaries of *B. cucurbitae* (Table 1). The rarefaction curves of all samples almost reached the saturation plateau (Fig. 2A), indicating that the sequencing results are sufficient to reflect the diversity contained in the current samples. Continuing to increase the sequencing depth did not increase the number of new undiscovered amplicon sequence variants (ASVs). The abundance grade curve (Fig. 2B) for all samples was almost straight, which reflects the small differences in abundance between ASVs in the community.

**Table 1:** Numbers of taxonomic categories of bacteria detected in eggs, primary ovaries, and mature ovaries of *Bactrocera cucurbitae*.

Sample	Phylum	Class	Order	Family	Genus	Species
Egg1	6	15	26	45	57	42
Egg2	7	14	25	37	46	26
Egg3	9	18	28	48	69	41
Egg4	7	12	25	35	42	23
Egg5	6	14	27	40	48	24
Egg6	7	13	27	41	53	32
OVI1	8	17	29	54	78	47
OVI2	7	12	18	29	31	15
OVI3	5	10	13	22	28	10
OVI4	6	15	25	39	43	21
OVI5	7	13	19	30	33	17
OVI6	11	20	28	43	60	28
OVII1	7	14	23	34	34	14
OVII2	8	14	22	31	35	15
OVII3	7	13	22	29	29	19
OVII4	5	10	18	29	29	16
OVII5	6	13	20	29	35	20
OVII6	6	14	20	29	30	15
Total	19	39	73	151	287	376

Egg1–egg6: Groups 1–6 samples were *B. cucurbitae* eggs. OVI1–OVI6: Groups 1–6 samples were *B. cucurbitae* primary ovaries. OVII1–OVII6: Groups 1–6 samples were *B. cucurbitae* mature ovaries.

### Microbiota in the eggs of *B. cucurbitae*

**Figure 3:**
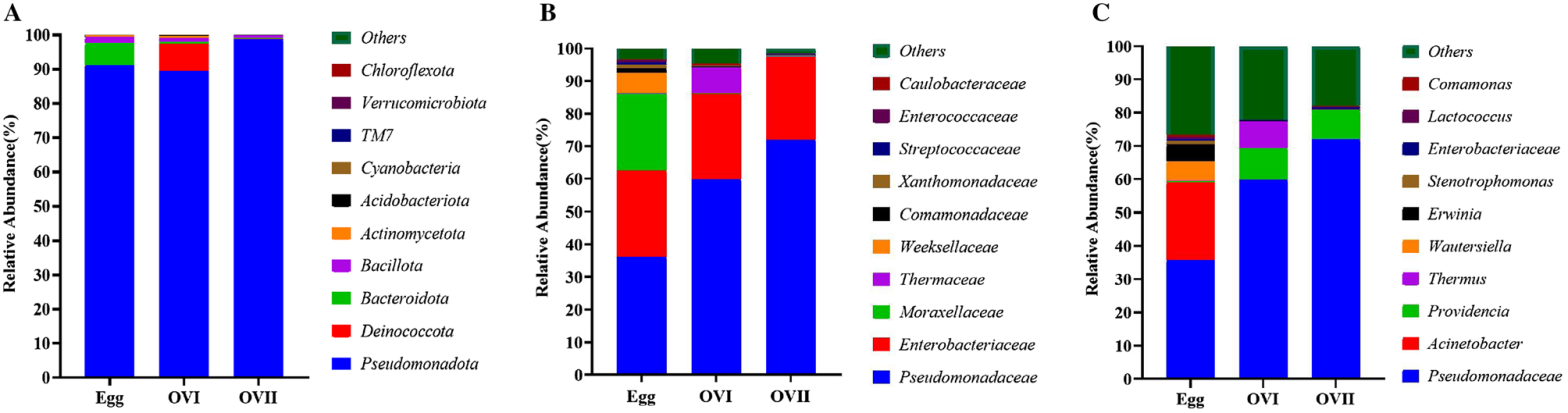
(**A**) Relative phylum-level abundances of microbial communities in *B. cucurbitae*. (**B**) Relative family-level abundances of microbial communities in *B. cucurbitae.* (**C**) Relative genus-level abundances of microbial communities in all samples. Egg: Eggs of *B. Cucurbitae*; OVI: Primary ovaries of *B. cucurbitae*; OVII: Mature ovaries of *B. cucurbitae*.

Pseudomonadota, Bacteroidota, Bacillota, Actinomycetota, and other bacterial phyla were detected in the eggs of *B. cucurbitae*. Pseudomonadota had the highest relative abundance (91.07%) (Fig. 3A). The relative abundances of Bacteroidota, Bacillota, Actinomycetota, and other bacterial phyla were 6.57%, 1.67%, 0.43%, and 0.26%, respectively (Fig. 3A). In total, we detected 74 bacterial families in the eggs of *B. cucurbitae* (Table 1). The two major bacterial families were Pseudomonadaceae and Enterobacteriaceae, with relative abundances of 36.06% and 26.43%, respectively (Fig. 3B). We detected 315 genera in the eggs of *B. cucurbitae* (Table 1), in which *Pseudomonas*, *Acinetobacter*, *Wautersiella, Erwinia, Stenotrophomonas,* and *Comamonas* were among the top 10 most abundant genera (Fig. 3C and Supplementary Table S4).

### Microbiota in the primary ovaries of *B. cucurbitae*

Pseudomonadota (89.45%), Deinococcota(8.06%), Bacteroidota(0.43%), Bacillota (1.14%), Acidobacteriota (0. 21%), and other bacterial phyla (0.72%) were detected in the primary ovaries of *B. cucurbitae* (Fig. 3A). We observed a total of 77 bacterial families (Table 1), among which Pseudomonadaceae and Enterobacteriaceae were predominant, with relative abundances of 59.80% and 26.30%, respectively (Fig. 3B). The relative abundances of *Pseudomonas*, *Providencia*, and *Thermus* were higher in the primary ovaries than those in the eggs. However, the opposite was true for *Acinetobacter*, *Wautersiella*, and *Erwinia* (Supplementary Table S4). The 10 most abundant bacterial genera were *Pseudomonas*, *Providencia*, *Thermus*, *Morganella*, *Brevundimonas*, *Leucobacter*, *Anoxybacillus*, *Paenibacillus, Dysgonomonas,* and other bacteria (Supplementary Table S4).

### Microbiota in the mature ovaries of *B. cucurbitae*

**Figure 4:**
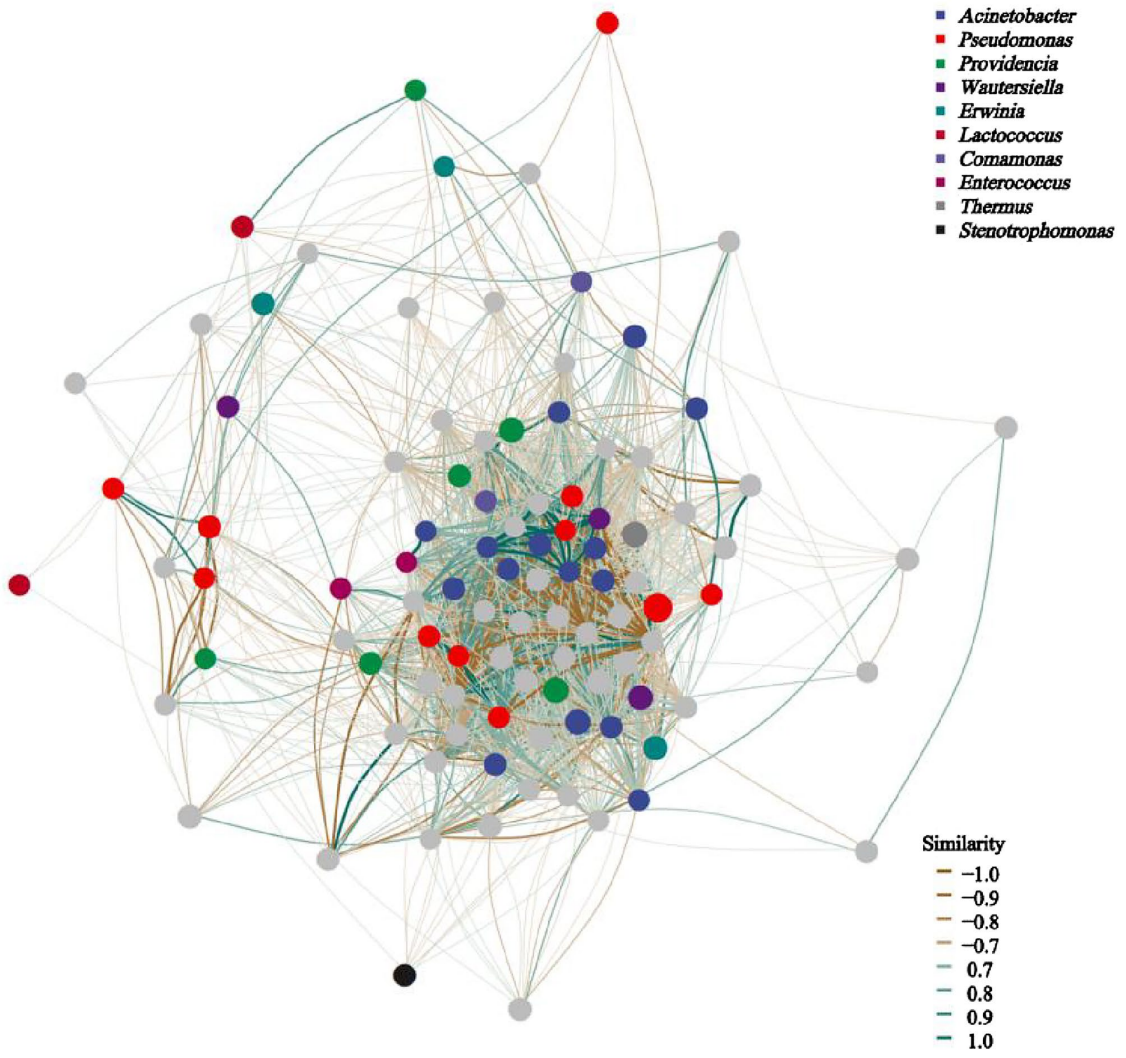
Association network. The nodes (node) represent the ASVs in the sample, for which the node size is directly proportional to its abundance (in log2 (CPM/n)), and identifies the 10 most nodes (module) with different colors. Lines between nodes (edge) indicate correlations between two connected nodes.

Pseudomonadota, Deinococcota, Bacteroidota, Bacillota, Actinomycetota, and other bacterial phyla were detected in the mature ovaries of *B. cucurbitae*. Pseudomonadota was the predominant phylum and had the highest relative abundance (98.83%) (Fig. 3A). The relative abundance of Bacillota was the highest (0.72%), followed by Deinococcota (0.14%), Bacteroidota (0.14%), Actinomycetota (0.12%), and other bacterial phyla (0.05%) (Fig. 3A). We detected 67 bacterial families (Table 1), among which Pseudomonadota and Enterobacteriaceae were predominant, with relative abundances of 72.03% and 25.43%, respectively (Fig. 3B). We detected 87 bacterial genera (Table 1), among which *Pseudomonas*, other bacteria, *Providencia*, *Lactococcus*, *Enterobacter, Erwinia, Thermus, Comamonas, Stenotrophomonas*, and *Acinetobacter* were the 10 most abundant genera (Supplementary Table S4). For all three sample categories, the common genera were *Pseudomonas, Acinetobacter, Providencia,* and *Enterobacter.* However, *Cronobacter* was not detected in the primary ovaries of *B. cucurbitae* (Supplementary Table S5). The bacterial networks of the 10 most abundant genera with the highest correlations are shown in (Fig. 4). The Spearman’s rank correlation between Acinetobacter and Pseudomonas was positive, suggesting a partnership between these genera.

### Comparisons among the microbial communities in eggs, primary ovaries, and mature ovaries

**Figure 5:**
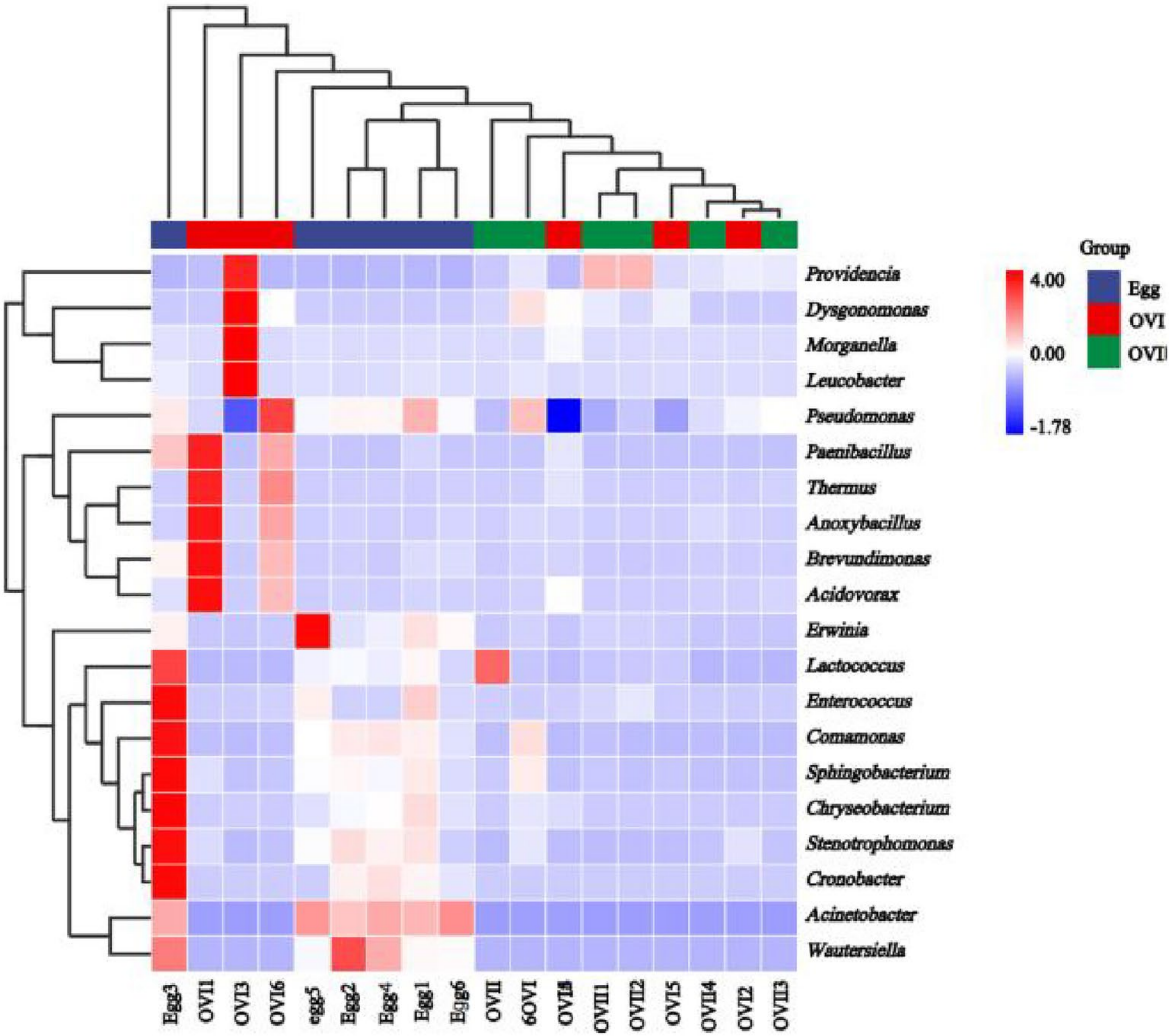
Heat map of species compositions. Heat map with high abundances at the genus level (sorted by abundance). The 20 most abundant genera were selected. UPGMA clustering was performed according to the Euclidean distance (Euclidean distance) of the species composition data and arranged according to the clustering results. The ordinate species were clustered using UPGMA clustering with the Pearson correlation coefficient matrix of their composition data and are arranged according to the clustering results. The legend shows the mean abundances. Unclassified genera are not shown.

The eggs exhibited a higher microbial diversity than those of the primary and mature ovaries. However, there were few differences between the microbial communities of the primary and mature ovaries. The major genera were *Pseudomonadaceae, Providencia,* and other bacteria (Fig. 3). To detect more species groups and compare them among the samples, community composition analysis was performed using heat maps. The 20 most abundant genera were selected for sorting (Fig. 5). *Pseudomonadaceae, Acinetobacter, Providencia,* and *Enterobacter* were observed consistently in all three *B. cucurbitae* sample types (Supplementary Table S5).

**Figure 6:**
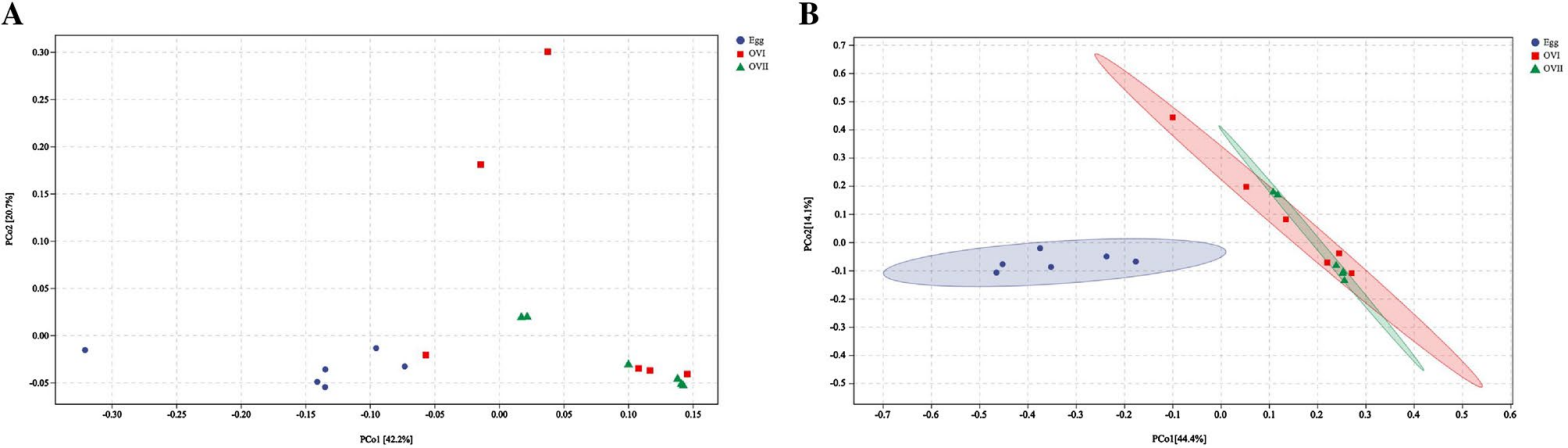
(**A**) Principal component analysis (PCoA) of bacterial communities in different tissues according to Weighted UniFrac distances. (**B**) PCoA of bacterial communities in different tissues according to Bray–Curtis distances. Egg: Eggs of *B. cucurbitae*; OVI: Primary ovaries of *B. Cucurbitae*; OVII: Mature ovaries of *B. cucurbitae*.

Principal coordinate analysis (PCoA) based on the Bray–Curtis distance and weighted UniFrac distance was used to compare the community similarities between samples. The PCoA scatter plot indicates that two characteristic values contributed to the largest differences between the samples, which had degrees of influence of 42.2% and 20.7% based on the weighted UniFrac distance (Fig. 6A), and 44.4% and 14.1% based on the Bray–Curtis distance, This suggests that the microbial community in the eggs is unique compared with those in the ovaries at different developmental stages (Fig. 6B). PERMANOVA analysis indicates that there were significant differences between the egg and ovary microbiota of *B. cucurbitae* (Table 2; Egg × OVI PERMANOVA: *p* = 0.003; Egg × OVII PERMANOVA: *p* = 0.003). However, there were no significant differences between the ovary microbiota during different developmental stages (Table 2; OVI × OVII PERMANOVA: *p* = 0.36).

**Table 2:** PERMANOVA results of the bacterial communities in eggs, primary ovaries, and mature ovaries of *B. cucurbitae*.

Group1	Group2	Sample size	Pseudo-F	*P*-value	q-value
all	–	18	5.869541	0.001	–
Egg	OVI	12	6.421584	0.003	0.0045
Egg	OVII	12	10.990284	0.003	0.0045
OVI	OVII	12	1.105677	0.36	0.36

PERMANOVA was generated using 999 permutations, and the individual repeat was included in the model as a random effect. Egg: Eggs of *B. cucurbitae*. OVI: Primary ovaries of *B. cucurbitae*. OVII: Mature ovaries of *B. cucurbitae.*

**Figure 7:**
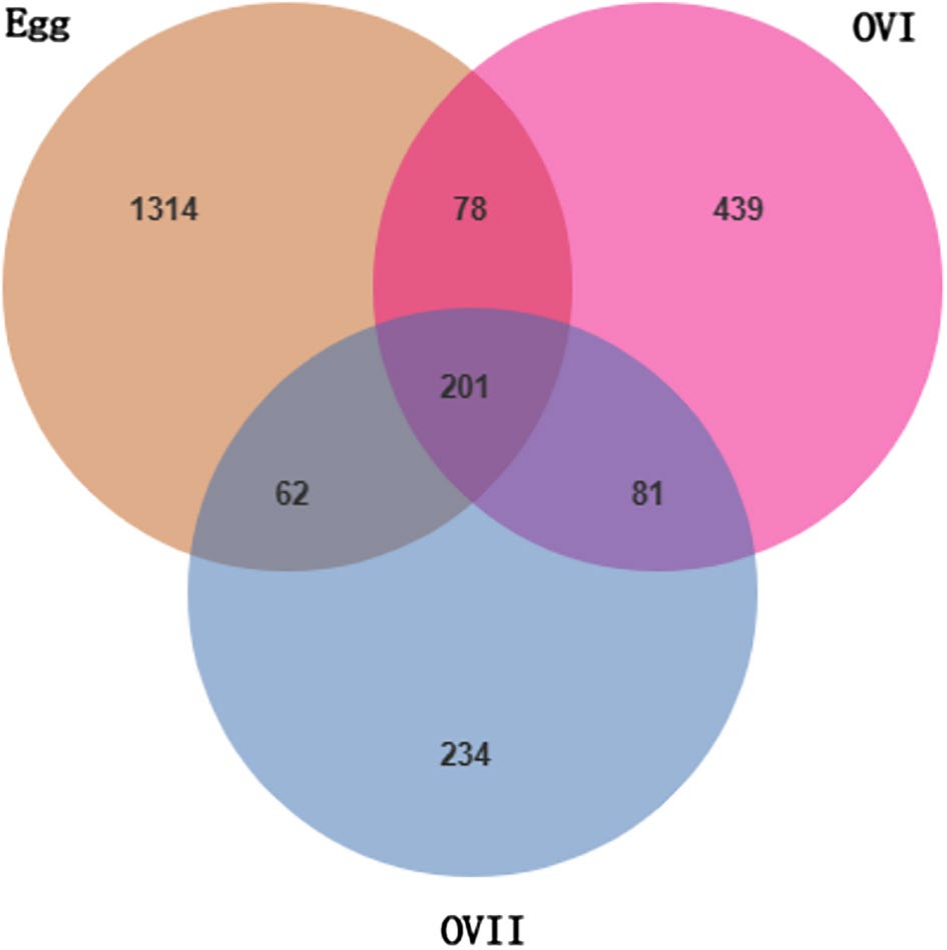
Venn diagram of bacterial community ASVs in *B. cucurbitae*. Egg: *B. cucurbitae* eggs; OVI: Primary ovaries of *B. cucurbitae*; OVII: Mature ovaries of *B. cucurbitae*.

Venn diagrams show that operational taxonomic units (ASVs) overlapped among the egg, primary ovary, and mature ovary bacteria in *B. cucurbitae* (Fig. 7). Specifically, 201 shared ASVs were observed, comprising 12.15%, 25.16%, and 34.78% of the bacteria in the eggs, primary ovaries, and mature ovaries, respectively (Fig. 7).

**Figure 8:**
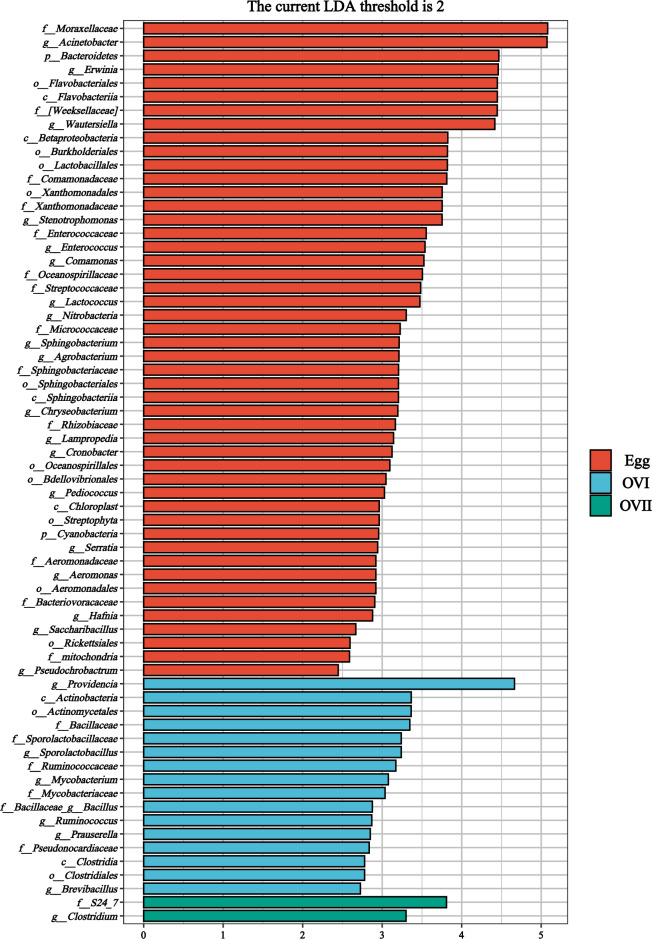
Bacterial communities with linear discriminant analysis (LDA) score > 2 in the eggs, primary ovaries, and mature ovaries of *B. cucurbitae*. The ordinate is taxa with significant differences between the groups, and the abscissa visually displays the LDA logarithmic scores for each taxon in a bar chart. Taxa are sorted by the size of the score to describe their specificity in the sample grouping. Longer lengths indicate more significant differences between the taxa, and the colors of the bars indicate the most abundant sample grouping for those taxa.

To investigate the significantly different biomarkers among the detected microbial communities, linear discriminant analysis effect size (LEfSe) was used to screen the genera among the groups based on a standard linear discriminant analysis (LDA) value > 2 (Fig. 8). The eggs had the widest microbial taxonomic diversity (LDA > 2), with 49 genera that mainly belonged to Moraxellaceae, Acinetobacter, Bacteroidota , Erwinia, Flavobacteriales, Flavobacteriia, Weeksellaceae, and Wautersiella. The primary ovaries had the second widest microbial taxonomic diversity, with 16 genera that mainly belonged to Providencia, Actinomycetota, Actinomycetales, Bacillaceae, Sporolactobacillaceae, and Sporolactobacillus. The mature ovaries had the lowest microbial taxonomic diversity (LDA > 2), with only two genera (S24_7 and Clostridium). LEfSe was also used to identify significant differences among the biomarkers in the samples (egg vs. OVI; egg vs. OVII) (Supplementary Fig. S1).

### Functional predictions of bacterial taxa in eggs, primary ovaries, and mature ovaries

**Figure 9:**
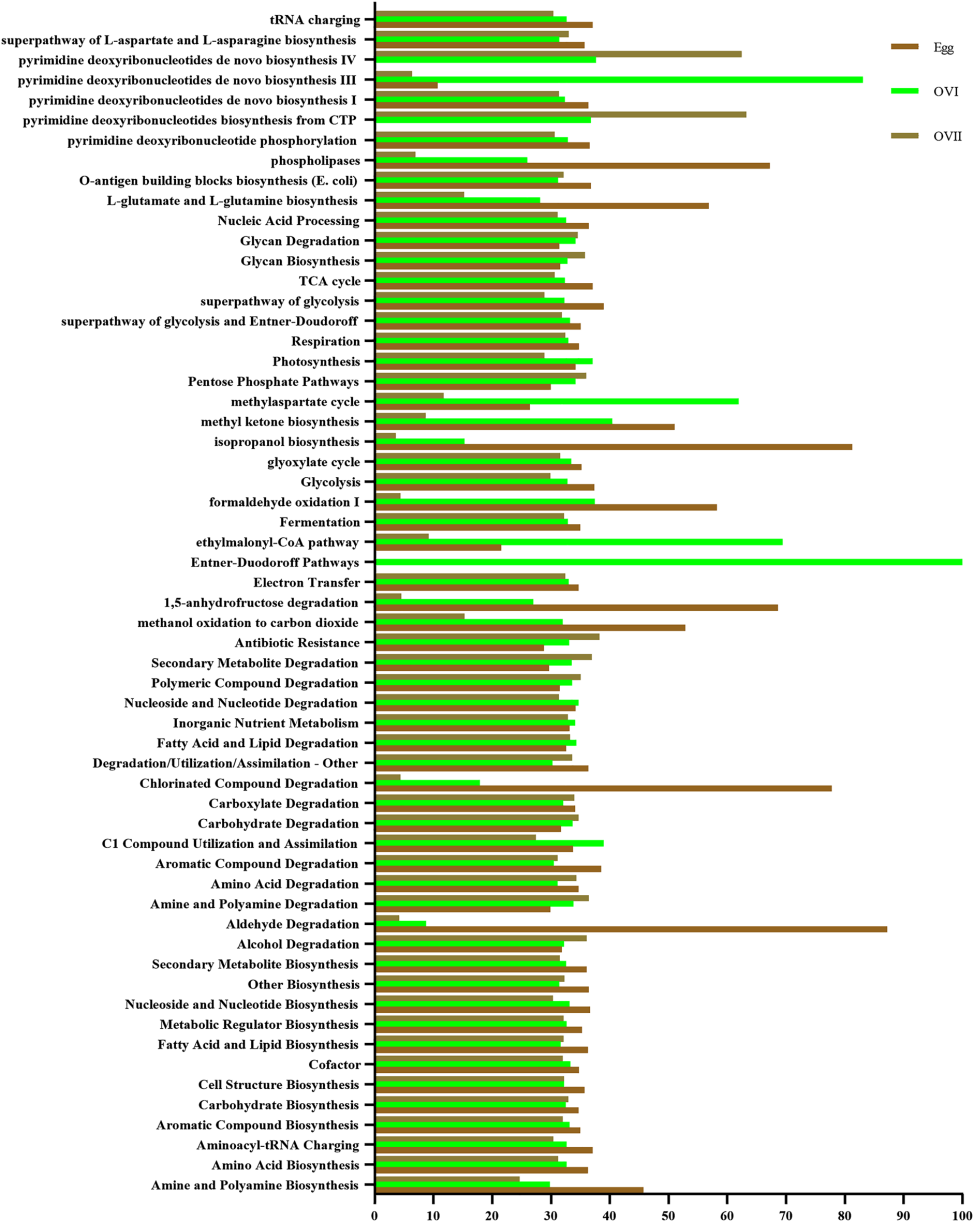
Metabolic pathways in bacterial communities in *B. cucurbitae* eggs, primary ovaries, and mature ovaries. KEGG functional secondary classifications at the genus level. The x-axis indicates the species, and the y-axis shows the relative abundance percentages of the metabolic pathways.

To clarify the roles of the microorganisms in the eggs, primary ovaries, and mature ovaries of *B.* cucurbitae, The metabolic function of the microbial flora is predicted by PPICRUSt2 (system genetic investigation of communities by reconstruction of unobobserved states) on the MetaCyc (https://metacyc.org/) and KEGG (https://www.kegg.jp/) databases, While screening the differential metabolic pathways between groups according to adjPvalues and logFC, And analyzed the species composition on the metabolic pathways (Fig. 9). Most of the bacterial taxa were functionally associated with biosynthesis (62.17%), degradation/utilization/assimilation (21.76%), precursor metabolite and energy generation (12.54%), and metabolic cluster formation (2.22%). In the biosynthesis category, cofactor, prosthetic group, electron carrier, and vitamin biosynthesis had the highest abundance (24.14%), followed by amino acid (22.11%), fatty acid and lipid (17.02%), nucleoside and nucleotide (16.70%), carbohydrate (7.26%), cell structure (5.49%), secondary metabolite (2.97%), aromatic compound (1.66%), other compound (0.84%), and amine and polyamine (0.63%) biosynthesis; aminoacyl-tRNA charging (0.63%); and metabolic regulator biosynthesis (0.57%). In the degradation/utilization/assimilation category, aromatic compound degradation had the highest abundance (15.17%), followed by secondary metabolite (14.49%), carbohydrate (12.21%), amino acid (11.14%), carboxylate (10.65%), and nucleoside and nucleotide (10.31%) degradation; inorganic nutrient metabolism (6.79%); amine and polyamine (5.59%), polymeric compound (3.62%), and fatty acid and lipid (3.54%) degradation; C1 compound utilization and assimilation (3.44%); and alcohol (1.13%) and aldehyde (0.17%) degradation (Supplementary Table S6).

## Discussion

Traditional isolation and culturing methods have provided limited information regarding bacteria in the melon fruit fly and other species^44^. Herein, we characterized the bacterial community in the eggs, primary ovaries, and mature ovaries of the melon fruit fly, *B. cucurbitae*. We used MiSeq sequencing, which allowed the microbial diversity to be investigated more than would be possible using conventional methods, to report the variations in microbial communities during different developmental stages. Pseudomonadota was the most abundant bacterial phylum in the eggs, primary ovaries, and mature ovaries of *B. cucurbitae*. The findings are similar to those of previous studies of the microbiota in *Ceratitis capitata*, *B. dorsalis*, *B. minax*, *B. cacuminata*, *B. tryoni*, *B. carambolae*, *B. neohumeralis*, *B. jarvisi*, *Anastrepha ludens*, *A. serpentine*, *A. striata*, and *A. obliqua*^23,26,45,46^. In this study, the developmental stage significantly affected the melon fly microbial diversity index. Chao1, observed-species, Simpson, and Shannon values, indicating that the microbial community richness varied during different developmental stages. Egg microbial richness was significantly higher than that of the ovaries, which may be the result of the unique physiological environment and function of the ovaries. Microbial diversity is also generally associated with food and environmental factors. Therefore, further studies are required to investigate the microbial diversity of melon flies across different hosts and geographical populations to more comprehensively determine the bacterial diversity.

Pseudomonadota was the predominant phylum in the *B. cucurbitae* eggs and ovaries and accounted for 98.83% of the microbiota in the mature ovaries. Similar findings have been obtained for other insects, including *B. minax* (Enderlein)^47^, *C. capitata* (Wiedemann)^48^, *Lutzomyia longipalpis*^49^, *Schistocerca gregaria*^50^, *Acyrthosiphon pisum*^51^, *Aphis fabae*^52^, and *Riptortus clavatus*^23^. In contrast, *Drosophila* spp. harbor bacteria belonging to Pseudomonadota and Bacillota, and the dominant commensal bacteria in *Musca domestica* belong to Bacillota and Bacteroides^53,54^. The major symbiotic bacteria in termites belong to Bacteroides, Bacillota, and Spirochaetes^55,56^, whereas the dominant bacterial symbionts in *Lymantria dispar, Helicoverpa armigera, Bombyx mori*, and *Plutella xylostella* belong primarily to Bacillota. The most abundant symbiotic bacteria in *Holotrichia glabripennis* belong to Pseudomonadota and Actinomycetes^57^. The diversity of bacterial symbionts in insects varies with diet and environmental factors^43^. Therefore, further investigation is necessary to determine the microbial diversities in the eggs and ovaries of different populations of *B. cucurbitae*. Of the bacterial genera identified throughout the developmental stages of *B. cucurbitae*, *Pseudomonadaceae* was the most abundant genus. This result was not in agreement with previous findings obtained for fruit fly microflora, which indicated that *Klebsiella* and *Citrobacter* were the most abundant genera^23^. For *C. capitata*, the most abundant bacterial genera were *Klebsiella*, *Pantoea*, *Enterobacter*, *Pectinobacter*, and *Citrobacter*^51^. The functions of these bacteria are worth examining. Detecting bacteria or specific genes related to the reproductive development of *B. cucurbitae* can also aid the development of microbially based control strategies.

*Pseudomonas* was the abundant bacterial genus in the eggs, primary ovaries, and especially the mature ovaries of *B. cucurbitae* (≥ 72.02%). In contrast, *Klebsiella* and *Citrobacter* are the main bacterial genera in *B. minax* (Enderlein). The most abundant symbiotic genera in *C. capitata* (Wiedemann) were *Klebsiella, Pantoea, Enterobacter, Pectobacterium*, and *Citrobacter*^51^. These symbionts might be implicated in the reproduction, growth, development, and biosynthesis of *B. cucurbitae*. Certain *Pseudomonas* spp. are harmful to insects, such as Pseudomonas *fluorescens*, which kills mosquitoes and *Musca domestica* L.^58^. *Pseudomonas aeruginosa* is pathogenic to *Caenorhabditis elegans*, *Drosophila* spp.*,* and *Hylesia metabus* larvae^59,60^ and shortens the lifespan of *C. capitata*^51^. However, certain *Pseudomonas* spp. are beneficial to insects, such as *P. aeruginosa*, which resists parasites in mosquitoes^61^, and a *Pseudomonas* species that produces the anti-tumor polysterin pederin in *Paederus fuscipes*^62^. Another *Pseudomonas* species is antagonistic to certain entomopathogenic fungi^63^. Further research is therefore needed to explore the physiological functions of *Pseudomonas* spp. in *B. cucurbitae*.

Previous studies demonstrated that the community composition of insect gut bacteria, mostly dominated by members belonging to phyla Pseudomonadota and Bacillota, followed by Bacteroidota , Actinomycetota, and Tenericutes, differs owing to variation in host location, food, developmental stage, physiology, and phylogeny^64^. Similarly, our results indicated that Pseudomonadota is the main phylum identified among the insect gut bacteria, and hence, the microbes present in the gut can be transmitted to the ovary and play an important role. Previous research detected that the *Enterobacteriaceae* community in the gut of medflies may indirectly contribute to host fitness by preventing the establishment or proliferation of pathogenic bacteria. Previously, Eyal Ben Ami demonstrated that the *Klebsiella oxytoca*-supplemented diets significantly shortened the mating latency of the sterile male *Mediterranean* fruit flies. Romero et al*.* demonstrated that *Citrobacter freundii* stimulated oviposition to its greatest extent and also supported stable fly development^22^. Gut bacteria that have vital roles in insect growth and development can be transferred from one generation to the next through infected ovaries. *Candidatus Riesia* sp. is localized in the gut of *Pediculus humanus* during the female development. The symbiont bacteria exit through a hole of the bacteriocytes and move posteriorly along the surface of the gut to the lateral fallopian tube, and subsequently, they reach and aggregate in the side fallopian tube and eggs^65^.

To date, several important Drosophila management strategies have been developed to control cash crops. Symbiodinium has become an important weapon for controlling Drosophila, For example, *Klebsiella oxytoca, K. pneumonia, Enterobacter cloacae, Pantoea agglomerans, Citrobacter sp., Providencia sp., Raoultella terrigena*, and *Bacillus cereus* have acted as attractants to control *B. dorsalis, Z. tau, B. cucurbitae*, *and B. zonata*. In addition, bacteria contribute substantially to SIT by increasing the calls, life expectancies, and mating abilities of mass-reared *C. capitata* male adult flies. Symbiotic bacteria also influence the mass rearing of parasitoids, as host detection has suggested that the combination of attractancy, SIT, and parasitoid application could be a suitable technique for controlling fruit fly species^42,63^. The present study provides new insights into the microbial community structure and its abundance during the different developmental stages of *B. cucurbitae*, and provides a theoretical basis for developing pest management strategies. The variations in the bacterial community throughout the developmental stages of *B. cucurbitae* may be the result of their habitat during these life stages. In addition to habitat, the transmission patterns of different bacterial species may affect their presence/absence during different life stages. The differences in the relative bacterial abundances in *B. cucurbitae* observed in this study may also be the result of geographical isolation. The diversity and abundance of the bacterial community can also vary with pH, partial oxygen pressure, and the presence/absence of physical barriers.

In summary, this is the first study to characterize the bacterial diversity, abundance, and functional predictions associated with the eggs and ovaries of *B. cucurbitae* using Illumina-based sequencing. We found that the alpha diversity indices differed significantly between the eggs and ovaries, but not between the primary and mature ovaries of *B. cucurbitae*. Overlapping bacterial taxa were detected in the ovaries and eggs. These taxa could have important roles in the growth and development of the host insect. The main roles of the commensal bacteria in *B. cucurbitae* included biosynthesis, degradation/utilization/assimilation, and precursor metabolite and energy generation. Further experiments should be performed to determine the functions of these bacteria. Overall, the findings of the present study provide a fine-scale understanding of the bacterial diversity in *B. cucurbitae*, which can be used to develop novel pest management strategies.

## Materials and methods

### Insects

Melon flies [*B. cucurbitae*] were collected from the Sugarcane Institute of the Guangxi Academy of Agricultural Sciences, Guangxi Zhuang Autonomous Region, southern China (22° 48′ N, 108° 22′ E, 79 m a.s.l.) in September 2022. The insects were sustained on a 1:2 (w/w) yeast powder:sugar feed culture at 26 ± 1 °C, 75–80% RH, and 14 h:10 h (light:dark) photoperiod. When the larvae reached maturity, they were placed in a sand cup at 60–70% RH. Newly emerged female flies were placed separately in 30 cm × 30 cm × 30 cm Perspex (Bio-agent acrylic) cages (Rescholar Equipment, Haryana, India) and provided with a controlled diet and water-soaked cotton.

### Egg collection

Eight days after adult emergence, bacterially uninfected cucumber slices were placed in each cage for adults to lay eggs. After 12 h, the cucumber slices were removed from the cage and the eggs were collected with a fine brush. The pooled egg samples were then washed with sterile ddH_2_O on a UV-sterilized workbench (AIRTECH JAPAN LTD., Tokyo, Japan) for 30 min. Finally, the eggs were stored in a 1.5 mL sterile tube at −80 °C for subsequent use. Two hundred eggs were pooled into one biological sample, and a total of six biological samples were obtained.

### Ovarian anatomy

Ovaries were dissected from emerged adults (matured in a home cage) on days 1 (primary ovaries) and 8 (mature ovaries). The ovaries were dissected on a UV-sterilized workbench. Whole adults were rinsed with sterile ddH_2_O, disinfected with 75% ethanol for 90 s, and rinsed again with sterile ddH_2_O. Before dissection, the adults were placed in a −20 °C refrigerator for 3 min. The adults were then dissected in phosphate-buffered saline (PBS; pH = 7; Biosharp Life Sciences, Hefei, Anhui, China) under a stereoscope (NIS-Elements, 20 ×, Shanghai, China). Adult worm-acquired ovaries were dissected and placed in a 1.5 mL sterile tube and stored at − 80 °C until use. Fifteen ovaries were pooled into one biological sample and a total of 12 biological samples were produced (adults on days 1 and 8 were dissected separately, with 6 biological samples each).

### 16S High-throughput sequencing

The samples were sent to Shanghai Personalbio Biotechnology Co., Ltd. for DNA extraction. The DNA was quantified, and Illumina sequencing was performed at Shanghai Personalbio Biotechnology Co., Ltd. DNA was extracted from all samples using a DNA extraction kit according to the manufacturer’s instructions (TIANGEN BIOTECH CO. LTD., Beijing, China) and verified using gel electrophoresis. The extracted DNA was amplified using the universal primer sets 338F (5′-ACTCCTACGGGAGGCAGCAG-3′) and 806R (5′-GGACTACHVGGGTWTCTAAT-3′), which targeted the V3–V4 region of the bacterial 16S ribosomal RNA (rRNA) genes. For all reactions, a 25 μL system with 25 ng DNA template, 12.5 μL PCR premix, and 2.5 μL forward and reverse primers were used and finally adjusted to 25 μL with ddH_2_O. The PCR amplification conditions were as follows: pre-denaturation (95 °C, 3 min), 30 cycles of amplification (95 °C denaturation for 30 s, 60 °C annealing for 30 s, and 72 °C extension for 45 s), and final extension at 72 °C for 10 min. The PCR products were examined on 1.5% agarose gel and purified using a QIAGEN Gel Extraction Kit (Qiagen, Dusseldorf, Germany). The purified products were sequenced using an Illumina MiSeq platform by Shanghai Personal Biotechnology Co., Ltd. (Shanghai, China). We performed 2 × 250 bp two-end sequencing using the MiSeq Reagent Kit V3 (600 cycles). After filtering out low quality or ambiguous reads and removing chimeric sequences, the high-quality sequences were clustered into ASVs at 100% similarity, and bacterial taxonomy was phylogenetically assigned using the Ribosomal Database Project classifier^66^.

### Data analysis

Microbiome information was analyzed using QIIME2 2019.4, with the processes modified and refined using official tutorial 4 (https://docs.qiime2.org/2019.4/tutorials/). Raw sequence data were processed using the demux plugin for decoding and cutadapt for primer excision. The data were then processed (i.e., quality filtering, denoising, and chimera removal) using the DADA2 plugin. The obtained sequences were merged based on 100% sequence similarity, and characteristic ASVs and abundance data tables were generated.

### Taxonomic composition analysis

The composition and abundance distribution tables for each sample at the phylum, family, and genus levels were obtained using the RStudio software package. Based on the comprehensive data, the 10 most abundant of each group were selected for analysis.

### Alpha diversity index analysis

First, using the QIIME2 software package, the total number of sequences for each sample in the ASV abundance matrix differed. Random sampling under depth and sparse curves were plotted with the number of sequences drawn at each depth and their corresponding ASV number to determine whether the sequencing depth of each sample was sufficient to reflect the microorganisms contained in the community sample diversity. Second, to compare the diversities of different samples to the sample with the fewest sequences in the whole sample, 95% of the sequences were randomly drawn in the ASV abundance matrix to correct the sequencing depth diversity differences. Subsequently, the Chao1, observed species, Shannon, and Simpson diversity indices were calculated separately for each sample using QIIME2. A boxplot was created to compare the richnesses and evenness of the The alpha diversity and relative abundance data were analyzed using one-way analysis of variance (ANOVA) with SPSS 26.0 (IBM SPSS Statistics), and multiple comparisons were analyzed using Tukey’s test.

### Beta diversity analysis

Beta diversity analysis was performed using the weighted UniFrac and Bray–Curtis distance metrics to investigate changes in the microbial community structure among the samples with the R and QIIME2 software packages, and was visualized using the PCoA method.

### Analysis of differences between groups at each taxonomic level

Permutational multivariate analysis of variance (PerMANOVA) was generated using 999 permutations, and the individual repeats were included in the model as a random effect. Using QIIME2, samples at the phyla, class, order, family, genus, and species levels, as well as abundance tables, were analyzed using PERMANOVA (Adonis/PERMANOVA analysis) to evaluate any significant differences in microbial community structure among the groups. The LEfSe method was used to detect the richness differences among the groups.

### Construction of association network and prediction of microbial metabolic functions

The association network was constructed using the SparCC analysis method, for which the pseudocount in SparCC was set to 10^–6^. The phases were determined by the random matrix theory-based approach of the R language RMThreshold data package. Relational number R values and significance (*P*) values were calculated. Based on the correlation coefficients and significance, we constructed a modular network using R Language and ggraph packets for a visual presentation of the associated network, in which nodes represent ASVs and wiring between the nodes represents correlations between the ASVs. The metabolic functions of microbial community were determined using PICRUSt2 (system genetic investigation of communities by reconstructing unobserved states) (Douglas, et al. preprint) with the MetaCyc (https://metacyc.org/) and KEGG (https://www.kegg.jp/) databases^67^. Based on the predicted results, the differential metabolic pathways between groups were screened using adjPvalues, logFC, and species composition of the metabolic pathway analysis to predict the PCoA at the functional level of the functional units.

## Supplementary Information

Supplementary Information.

## Data Availability

The raw sequence data obtained in this study were deposited in the Sequence Read Archive (SRA) of the National Center for Biotechnology Information (NCBI) under accession number PRJNA937695. All authors approved the disclosure of data because this study did not involve human subjects.
