# Supplementary material for: Comparison of bacterial diversity in Bactrocera cucurbitae (Coquillett) ovaries and eggs based on 16S rRNA sequencing
Source: Sci Rep. 2023 Jul 21;13:11793. doi: 10.1038/s41598-023-38992-z (PMC10362026; doi:10.1038/s41598-023-38992-z)
Supplement: Supplementary file 1 — Supplementary Information. [file 41598_2023_38992_MOESM1_ESM.docx]

| Sample | Input | Filtered | Denoised | Merged | Non-chimeric | Non-singleton |
| --- | --- | --- | --- | --- | --- | --- |
| Egg1 | 141060 | 119943 | 118864 | 117901 | 90924 | 90632 |
| Egg2 | 148207 | 126934 | 126005 | 125093 | 92612 | 92377 |
| Egg3 | 133704 | 116730 | 115521 | 113934 | 79942 | 79405 |
| Egg4 | 134766 | 114578 | 113401 | 112424 | 78868 | 78668 |
| Egg5 | 136792 | 120045 | 119001 | 118146 | 82449 | 82203 |
| Egg6 | 133248 | 113125 | 112312 | 111706 | 92775 | 92673 |
| OVI1 | 143513 | 121048 | 120320 | 119543 | 109797 | 109665 |
| OVI2 | 140255 | 115491 | 114853 | 114474 | 104637 | 104597 |
| OVI3 | 135475 | 118270 | 117731 | 117362 | 88224 | 88204 |
| OVI4 | 145633 | 115868 | 115344 | 115081 | 113502 | 113492 |
| OVI5 | 145083 | 116860 | 116223 | 115899 | 106400 | 106377 |
| OVI6 | 138662 | 119143 | 118223 | 117508 | 96303 | 96168 |
| OVII1 | 143208 | 121700 | 121153 | 120782 | 96645 | 96610 |
| OVII2 | 144618 | 122047 | 121512 | 121101 | 97595 | 97550 |
| OVII3 | 145815 | 116003 | 115555 | 115278 | 109926 | 109906 |
| OVII4 | 143847 | 115848 | 115283 | 115017 | 110279 | 110247 |
| OVII5 | 139463 | 113514 | 113058 | 112645 | 102185 | 102151 |
| OVII6 | 139916 | 110581 | 110164 | 109945 | 106448 | 106437 |
| Total | 2533265 | 2117728 | 2104523 | 209839 | 1759511 | 1757362 |

**SUPPLEMENTARY TABLE S1. High-throughput sequencing and ASVs for *Bactrocera cucurbitae* eggs, primary ovaries, and mature ovaries.**

Egg1–egg6: Groups 1–6 samples were *Bactrocera cucurbitae* eggs. OVI1–OVI2: Groups 1–6 samples were *Bactrocera cucurbitae* primary ovaries. OVII1–OVII6: Groups 1–6 samples were *Bactrocera cucurbitae* mature ovaries.

**SUPPLEMENTARY TABLE S2. Alpha diversity indices of *Bactrocera cucurbitae* eggs, primary ovaries, and mature ovaries.**

| **sample** | **ASV number** | **Chao1** | **Simpson** | **Shannon** | **Observed-species** |
| --- | --- | --- | --- | --- | --- |
| Egg1  Egg2  Egg3 | 591  606  767 | 622.169  636.179  782.622 | 0.8433  0.899806  0.955111 | 5.12578  5.51109  6.27746 | 591.1  602.1  767.8 |
| Egg2 | 606 | 636.179 | 0.899806 | 5.51109 | 602.1 |
| Egg3 | 767 | 782.622 | 0.955111 | 6.27746 | 767.8 |
| Egg4 | 589 | 609.25 | 0.949741 | 6.0991 | 590.8 |
| Egg5 | 538 | 555.315 | 0.890878 | 4.96608 | 534.6 |
| Egg6 | 434 | 462.086 | 0.785565 | 4.20969 | 435.8 |
| OVI1 | 389 | 411.918 | 0.66475 | 2.81752 | 389.1 |
| OVI2 | 238 | 249.304 | 0.459291 | 2.28115 | 237.8 |
| OVI3 | 140 | 146.161 | 0.777214 | 2.94153 | 138.3 |
| OVI4 | 154 | 170.176 | 0.196678 | 0.997591 | 158.8 |
| OVI5 | 179 | 181.135 | 0.402325 | 1.85345 | 176.3 |
| OVI6 | 330 | 339.428 | 0.81128 | 3.94974 | 329.7 |
| OVII1 | 171 | 177.006 | 0.748972 | 3.2414 | 168.8 |
| OVII2 | 179 | 191.756 | 0.758774 | 3.2873 | 178.4 |
| OVII3 | 196 | 202.953 | 0.252646 | 1.31415 | 192.4 |
| OVII4 | 195 | 206.005 | 0.238185 | 1.25511 | 194.7 |
| OVII5 | 211  606  767 | 223.321 | 0.427623 | 2.18057 | 211.4 |
| OVII6 | 165 | 175.912 | 0.21922 | 1.13841 | 167.4 |

Egg1–egg6: Groups 1–6 samples were *Bactrocera cucurbitae* eggs. OVI1–OVI2:Groups 1–6 samples were *Bactrocera cucurbitae* primary ovaries. OVII1–OVII6: Groups 1–6 samples were *Bactrocera cucurbitae* mature ovaries.

**SUPPLEMENTARY TABLE S3. ANOVA of bacterial communities in *Bactrocera cucurbitae* eggs, primary ovaries, and mature ovaries.**

| **Factor** | **Chao1** | | **Simpson** | | **Shannon** | | **Observed-species** | |
| --- | --- | --- | --- | --- | --- | --- | --- | --- |
|  | F | *P* | F | *P* | F | *P* | F | *P* |
| **Eggs/primary ovaries** | 35.145 | <0.001 | 10.932 | 0.008 | 31.115 | <0.001 | 33.217 | <0.001 |
| **Eggs/mature ovarian** | 90.426 | <0.001 | 17.449 | 0.002 | 41.169 | <0.001 | 80.588 | <0.001 |
| **primaryovaries/ mature ovarian** | 1.487 | 0.251 | 0.606 | 0.454 | 0.485 | 0.502 | 1.587 | 0.236 |

**SUPPLEMENTARY TABLE S4. Relative abundances (%) of bacterial genera in *Bactrocera cucurbitae* eggs, primary ovaries, and mature ovaries.**

| **Genus** | **Sample** | | |
| --- | --- | --- | --- |
|  | **Egg** | **OVI** | **OVII** |
| *Pseudomonadaceae* | 35.7142 | 59.7835 | 72.0167 |
| *Acinetobacter* | 23.3037 | 0.1306 | 0.0685 |
| *Providencia* | 0.4212 | 9.4272 | 8.7521 |
| *Thermus* | 0.0761 | 7.9963 | 0.1373 |
| *Wautersiella* | 5.7968 | 0.0070 | 0.0007 |
| *Erwinia* | 5.0699 | 0.0669 | 0.1866 |
| *Stenotrophomonas* | 1.2347 | 0.0947 | 0.0707 |
| *Enterobacteriaceae* | 0.3822 | 0.2999 | 0.3111 |
| *Lactococcus* | 0.6055 | 0.0238 | 0.3534 |
| *Comamonas* | 0.6824 | 0.0061 | 0.1001 |
| *Enterococcus* | 0.6776 | 0.0067 | 0.0313 |
| *Morganella* | 0.0109 | 0.6011 | 0.0000 |
| *Brevundimonas* | 0.0765 | 0.4953 | 0.0128 |
| *Leucobacter* | 0.0277 | 0.4684 | 0.0085 |
| *Paenibacillus* | 0.1010 | 0.3576 | 0.0101 |
| *Dysgonomonas* | 0.0010 | 0.3207 | 0.0745 |
| *Anoxybacillus* | 0.0061 | 0.3641 | 0.0099 |
| *Sphingobacterium* | 0.3260 | 0.0133 | 0.0398 |
| *Chryseobacterium* | 0.3201 | 0.0077 | 0.0095 |
| *Cronobacter* | 0.2690 | 0.0000 | 0.0000 |
| Others | 24.8973 | 19.5290 | 17.8065 |

Egg*: Bactrocera cucurbitae* eggs. OVI: Primary ovaries of *Bactrocera cucurbitae.* OVII: Mature ovaries of *Bactrocera cucurbitae*. Data are means.

| **Sample** | **Genera(%)** |
| --- | --- |
| **Egg** | Pseudomonadaceae(35.7142) |
|  | Acinetobacter(23.3037) |
|  | Providencia(0.4212) |
|  | Enterobacteriaceae(0.3822) |
|  | Cronobacter(0.2690) |
| **0VI** | Pseudomonadaceae(59.7835) |
|  | Acinetobacter(0.1306) |
|  | Providencia(9.4272) |
|  | Enterobacteriaceae(0.2999) |
|  | Cronobacter(0.0000) |
| **0VII** | Pseudomonadaceae(72.0167) |
|  | Acinetobacter(0.0685) |
|  | Providencia(8.7521) |
|  | Enterobacteriaceae(0.3111) |
|  | Cronobacter(0.0000) |

**SUPPLEMENTARY TABLE S5. Stable bacterial genera in eggs, primary ovaries, mature ovaries, single tissues, or absence of tissue.**

Egg: *Bactrocera cucurbitae* eggs; OVI: Primary ovaries of *Bactrocera cucurbitae*; OVII: Mature ovaries of *Bactrocera cucurbitae*.

**SUPPLEMENTARY TABLE S6. Metabolic pathways of bacterial communities in *Bactrocera cucurbitae* eggs, primary ovaries, and mature ovaries.**

| Level1 | Level2 | Abundance |
| --- | --- | --- |
| Biosynthesis | Amine and Polyamine Biosynthesis | 887.29 |
| Biosynthesis | Amino Acid Biosynthesis | 31151.28 |
| Biosynthesis | Aminoacyl-tRNA Charging | 883.25 |
| Biosynthesis | Aromatic Compound Biosynthesis | 2333.7 |
| Biosynthesis | Carbohydrate Biosynthesis | 10226.64 |
| Biosynthesis | Cell Structure Biosynthesis | 7731.22 |
| Biosynthesis | Cofactor, Prosthetic Group, Electron Carrier, and Vitamin Biosynthesis | 34011.13 |
| Biosynthesis | Fatty Acid and Lipid Biosynthesis | 23977.35 |
| Biosynthesis | Metabolic Regulator Biosynthesis | 801.27 |
| Biosynthesis | Nucleoside and Nucleotide Biosynthesis | 23533.88 |
| Biosynthesis | Other Biosynthesis | 1182.23 |
| Biosynthesis | Secondary Metabolite Biosynthesis | 4189.29 |
| Degradation/Utilization/Assimilation | Alcohol Degradation | 551.06 |
| Degradation/Utilization/Assimilation | Aldehyde Degradation | 84.72 |
| Degradation/Utilization/Assimilation | Amine and Polyamine Degradation | 2714.43 |
| Degradation/Utilization/Assimilation | Amino Acid Degradation | 5413.01 |
| Degradation/Utilization/Assimilation | Aromatic Compound Degradation | 7368.32 |
| Degradation/Utilization/Assimilation | C1 Compound Utilization and Assimilation | 1671.48 |
| Degradation/Utilization/Assimilation | Carbohydrate Degradation | 5931.65 |
| Degradation/Utilization/Assimilation | Carboxylate Degradation | 5173.39 |
| Degradation/Utilization/Assimilation | Chlorinated Compound Degradation | 0.3 |
| Degradation/Utilization/Assimilation | Degradation/Utilization/Assimilation - Other | 841.16 |
| Degradation/Utilization/Assimilation | Fatty Acid and Lipid Degradation | 1718.75 |
| Degradation/Utilization/Assimilation | Inorganic Nutrient Metabolism | 3297.21 |
| Degradation/Utilization/Assimilation | Nucleoside and Nucleotide Degradation | 5008.13 |
| Degradation/Utilization/Assimilation | Polymeric Compound Degradation | 1760.36 |
| Degradation/Utilization/Assimilation | Secondary Metabolite Degradation | 7038.52 |
| Detoxification | Antibiotic Resistance | 749.46 |
| Detoxification | methanol oxidation to carbon dioxide | 10.3 |
| Generation of Precursor Metabolite and Energy | 1,5-anhydrofructose degradation | 0.84 |
| Generation of Precursor Metabolite and Energy | Electron Transfer | 2266.03 |
| Generation of Precursor Metabolite and Energy | Entner-Duodoroff Pathways | 0.01 |
| Generation of Precursor Metabolite and Energy | ethylmalonyl-CoA pathway | 1.72 |
| Generation of Precursor Metabolite and Energy | Fermentation | 7271.92 |
| Generation of Precursor Metabolite and Energy | formaldehyde oxidation I | 27.49 |
| Generation of Precursor Metabolite and Energy | Glycolysis | 2344.53 |
| Generation of Precursor Metabolite and Energy | glyoxylate cycle | 797.35 |
| Generation of Precursor Metabolite and Energy | isopropanol biosynthesis | 14.73 |
| Generation of Precursor Metabolite and Energy | methyl ketone biosynthesis | 14.85 |
| Generation of Precursor Metabolite and Energy | methylaspartate cycle | 5.35 |
| Generation of Precursor Metabolite and Energy | Pentose Phosphate Pathways | 3006.61 |
| Generation of Precursor Metabolite and Energy | Photosynthesis | 1171.05 |
| Generation of Precursor Metabolite and Energy | Respiration | 2292.67 |
| Generation of Precursor Metabolite and Energy | superpathway of glycolysis and Entner-Doudoroff | 846.54 |
| Generation of Precursor Metabolite and Energy | superpathway of glycolysis, pyruvate dehydrogenase, TCA, and glyoxylate bypass | 842.11 |
| Generation of Precursor Metabolite and Energy | TCA cycle | 7522.72 |
| Glycan Pathways | Glycan Biosynthesis | 704.66 |
| Glycan Pathways | Glycan Degradation | 848.69 |
| Macromolecule Modification | Nucleic Acid Processing | 1401.33 |
| Metabolic Clusters | L-glutamate and L-glutamine biosynthesis | 439.57 |
| Metabolic Clusters | O-antigen building blocks biosynthesis (E. coli) | 815.26 |
| Metabolic Clusters | phospholipases | 0.1 |
| Metabolic Clusters | pyrimidine deoxyribonucleotide phosphorylation | 817.07 |
| Metabolic Clusters | pyrimidine deoxyribonucleotides biosynthesis from CTP | 0.09 |
| Metabolic Clusters | pyrimidine deoxyribonucleotides de novo biosynthesis I | 943.8 |
| Metabolic Clusters | pyrimidine deoxyribonucleotides de novo biosynthesis III | 76.04 |
| Metabolic Clusters | pyrimidine deoxyribonucleotides de novo biosynthesis IV | 0.06 |
| Metabolic Clusters | superpathway of L-aspartate and L-asparagine biosynthesis | 1046.85 |
| Metabolic Clusters | tRNA charging | 883.25 |

Level 1: Layer 1 pathway. Level 2: Layer 2 pathway.

**SUPPLEMENTARY FIGURE S1. Metabolic pathway analysis plot.**
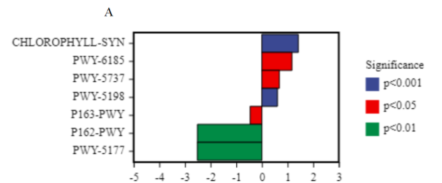

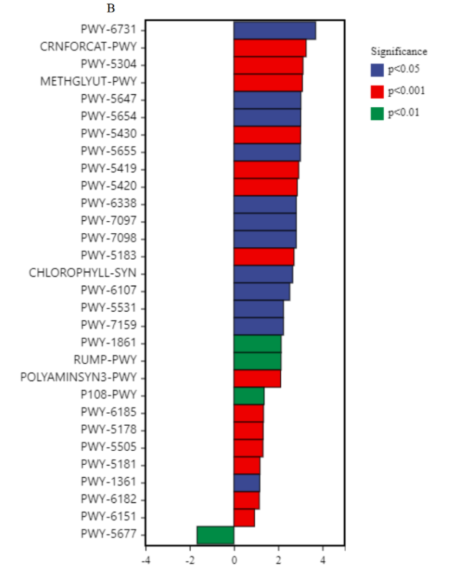


Analysis results were based on 7.1 (PICRUSt2 analysis). The positive value of the horizontal axis logFC (log2 (fold change)) represents the up-regulated group and the control group with the negative value; the ordinate is different pathway labels; and the significance is displayed in different colors.

(A) Egg (upward adjustment group) vs. OVI (control group). (B) Egg (upward adjustment group) vs. OVII (control group). Color reflects relevance or importance.
